# College Course About Flourishing and Students’ Mental Health During SARS-CoV-2

**DOI:** 10.1001/jamanetworkopen.2024.44845

**Published:** 2024-11-13

**Authors:** Matthew J. Hirshberg, Blake A. Colaianne, Mark T. Greenberg, Karen Kurotsuchi Inkelas, Richard J. Davidson, David Germano, John D. Dunne, Robert W. Roeser

**Affiliations:** 1 Center for Healthy Minds, University of Wisconsin, Madison; 2 Human Development and Family Studies, The Pennsylvania State University, University Park; 3 School of Education and Human Development, University of Virginia, Charlottesville; 4 Department of Religious Studies, University of Virginia, Charlottesville

## Abstract

This cohort study examines the association of a college course designed to strengthen skills for mental health taken in 2018 or 2019 with measures of anxiety, depression, and flourishing during the early months of the SARS-CoV-2 pandemic in 2020.

## Introduction

Adolescent mental health has been declining for decades, reaching a new low during the SARS-CoV-2 pandemic lockdown in Spring 2020.^1,2^ Entering college is an additional risk factor for internalizing symptoms and reduced flourishing.^1^ In 2016, scholars developed the Art and Science of Human Flourishing (ASHF), which is a general education college course designed to support adolescents and young adults transitioning to college by blending academic learning about flourishing with experiential meditation laboratories in which students learn practices (eg, mindfulness, self-inquiry) thought to strengthen skills associated with mental health.

A propensity score–matched (PSM) quasi-experimental trial conducted in Fall 2018 and 2019 semesters at 3 US universities reported multiple end-of-ASHF course benefits, including reduced depressive symptoms and increased flourishing.^3^ We recontacted these participants 5 or 17 months later during SARS-CoV-2 pandemic lockdown in Spring 2020 to test whether the ASHF was associated with reduced anxiety, reduced depression, and/or increased flourishing.

## Methods

In April to May 2020, we asked the 651 prior trial participants (ASHF = 217; PSM controls = 434) to participate in research on well-being during the SARS-CoV-2 lockdown. Approximately 46% (296 of 651) electronically consented to participate and enrolled. Compensation varied by university (eg, $10 gift card; lottery for 1 of 5 $200 checks). Each university obtained ethics board approval for all study procedures and materials. Reporting follows the STROBE guideline for cohort studies.

We assessed anxiety and depressive symptoms with the Generalized Anxiety Disorder-7^4^ and Patient Health Questionnaire-8,^5^ respectively, and flourishing with the Pemberton Happiness Index.^6^ On all measures, higher scores represent greater endorsement of the construct.

We checked for group and respondent/nonrespondent balance by estimating standardized differences on 23 variables collected at baseline (Table). Missing data were addressed by imputing 50 complete datasets using multiple imputation by chained equations (eAppendix in Supplement 1). On each complete dataset, we estimated a mixed-effects model that regressed the outcome at follow-up onto group (PSM control, ASHF), gender (woman, man), race (African American, Asian or Pacific Islander, Hispanic, Middle Eastern combined; White), the 8 baseline variables with absolute standardized group differences of at least 0.10 (Table), and baseline outcome score, with a random effect nesting participants within group, cohort (2018 or 2019), and university (university 1, university 2, or university 3; 12 level-2 clusters), pooling results (eAppendix in Supplement 1). We calculated the standardized mean difference (SMD) and its 95% CI by dividing the coefficient for group by the outcome’s baseline SD. We tested whether cohort moderated ASHF outcomes through an interaction term between cohort and group. Statistical significance was a 2-sided *P* < .05.

**Table.** Sample Characteristics and Baseline Balance Check

Characteristic	Participants, No. (%)			Standardized difference (95% CI)	
	Total (N = 296)	ASHF	Control	Group	Responders
Cohort					
2018	148 (50)	37 (35)	111 (58)	0.27 (0.13 to 0.42)	0.17 (0.03 to 0.32)
2019	148 (50)	68 (46)	80 (54)		
Gender^a^					
Women	225 (76)	79 (75)	146 (76)	0.01 (−0.14 to 0.15)	0.13 (−0.01 to 0.30)
Men	71 (24)	26 (25)	45 (24)		
Race^b^					
African American or Black	20 (7)	6 (6)	14 (7)	0.01 (−0.2 to 0.2)	0.01 (−0.14 to 0.15)
Asian or Pacific Islander	39 (13)	14 (13)	25 (13)		
Hispanic or Latino	14 (5)	6 (6)	8 (4)		
White	200 (68)	70 (67)	130 (68)		
Prefer not to say	23 (8)	9 (9)	14 (7)		
Living situation during SARS-CoV-2				0.28 (0.05 to 0.51)	NA^c^
Alone	129 (44)	54 (51)	75 (39)		
Roommates	65 (22)	23 (22)	42 (22)		
Family	28 (9)	8 (8)	20 (11)		
Other	73 (35)	19 (18)	54 (28)		
Outcome					
Anxiety symptoms, GAD-7 score, mean (SD)					
Baseline	6.90 (5.22)	7.30 (5.39)	6.71 (5.15)	0.11 (−0.15 to 0.38)	0.19 (−0.06 to 0.43)
SARS-CoV-2 follow-up	6.91 (5.66)	5.95 (4.78)	7.43 (6.03)		
Depressive symptoms, PHQ-8 score, mean (SD)					
Baseline	7.13 (5.56)	7.87 (6.05)	8.11(6.38)	−0.04 (−0.28 to 0.20)	0.11 (−0.05 to 0.28)
SARS-CoV-2 Follow-up	8.19 (5.87)	7.03 (5.22)	8.98 (6.20)		
Flourishing, score, mean (SD)					
Baseline	7.22 (1.57)	7.18 (1.56)	7.24 (1.59)	−0.04 (−0.28 to 0.21)	−0.03 (−0.20 to 0.13)
SARS-CoV-2 follow-up	7.38 (1.48)	7.65 (1.44)	7.23 (1.49)		
Baseline balance check					
Age	18.63 (0.61)	18.64 (0.68)	18.64 (0.56)	0.02 (−0.22 to 0.26)	−0.39 (−0.61 to −0.16)
Attention function	6.84 (1.23)	6.66 (1.24)	6.94 (1.22)	−0.23 (−0.47 to 0.01)	−0.15 (−0.31 to 0.01)
Attention regulation	2.94(0.91)	2.80 (0.94)	3.02 (0.88)	−0.24 (−0.48 to 0.0)	−0.17 (−0.34 to −0.01)
Common humanity	3.73 (0.81)	3.71 (0.73)	3.74 (0.85)	−0.04 (−0.27 to 0.20)	0.07 (−0.10 to 0.23)
Compassionate roommate goals	4.20 (0.62)	4.19 (0.55)	4.20 (0.66)	−0.03 (−0.27 to 0.22)	0.07 (−0.10 to 0.23)
Empathic concern	4.10 (0.74)	4.11 (0.75)	4.09 (0.74)	0.02 (−0.22 to 0.26)	0.06 (−0.11 to 0.22)
First-year undergraduate^d^	272 (92)	94 (90)	179 (94)	0.04 (−0.03 to 0.11)	0.08 (−0.07 to 0.23)
Healthy emotionality	4.63 (0.75)	4.41 (0.75)	4.70 (0.74)	−0.40 (−0.79 to 0.0)	0.01 (−0.23 to 0.26)
International student^d^	8 (8)	4 (4)	8 (4)	0.0 (−0.11 to 0.10)	0.01 (−0.14 to 0.15)
Mindfulness	3.08 (0.42)	2.98 (0.43)	3.14 (0.41)	−0.37 (−0.6 to −0.1)	−0.12 (−0.28 to 0.04)
Perspective taking	3.74 (0.80)	3.67 (0.79)	3.79 (0.80)	−0.15 (−0.39 to 0.09)	0.02 (−0.14 to 0.18)
Search for meaning	3.91 (0.98)	4.07 (0.89)	3.81 (1.02)	0.27 (−0.01 to 0.5)	0.10 (−0.06 to 0.27)
Self-compassion	2.82 (0.64)	2.75 (0.67)	2.86 (0.62)	−0.17 (−0.42 to 0.07)	−0.13 (−0.29 to 0.04)
Social awareness	4.24 (0.70)	4.23 (0.67)	4.25 (0.72)	−0.03 (−0.27 to 0.21)	0.07 (−0.10 to 0.23)
Stress	3.37 (0.84)	3.43 (0.83)	3.34 (0.84)	0.11 (−0.13 to 0.34)	0.39 (0.23 to 0.55)
Sleep quality	2.87 (0.59)	2.89 (0.54)	2.86 (0.62)	0.05 (−0.21 to 0.30)	0.23 (−0.01 to 0.47)

Abbreviation: ASHF, Art and Science of Human Flourishing participants; Control, propensity-score matched control participants.

^a^ All follow-up responders self-reported their gender as either women or men.

^b^ American Indian or Alaskan Native was included as a race/ethnicity category but was not endorsed by any participant. For analyses, a dichotomized race variable (African American, Asian/Pacific Islander, Hispanic, Middle Eastern combined; White) was used.

^c^ There is no nonrespondent data on this measure to allow a comparison between responders and nonresponders.

^d^ No. (%). See eAppendix in Supplement 1 for references of baseline balance check measures.

## Results

Among 296 participants included, 225 (76%) self-reported as women; 20 (7%) were African American or Black, 39 (13%) were Asian or Pacific Islander, 14 (5%) were Hispanic or Latino, and 200 (68%) were White (Table). Prior ASHF participation was associated with significantly lower anxiety (β [SE], −1.63 [0.60]; *P* = .007; SMD, −0.31 [95% CI, −0.55 to −0.07]) and depressive symptoms (β [SE], −1.97 [0.78]; *P* = .01; SMD, −0.32 [95% CI, −0.56 to −0.08]), and significantly higher flourishing (β [SE] 0.43, [0.17]; *P* = .01; SMD, 0.28 [95% CI, 0.04 to 0.52]) (Figure). ASHF outcomes were not moderated by study year.

**Figure.**
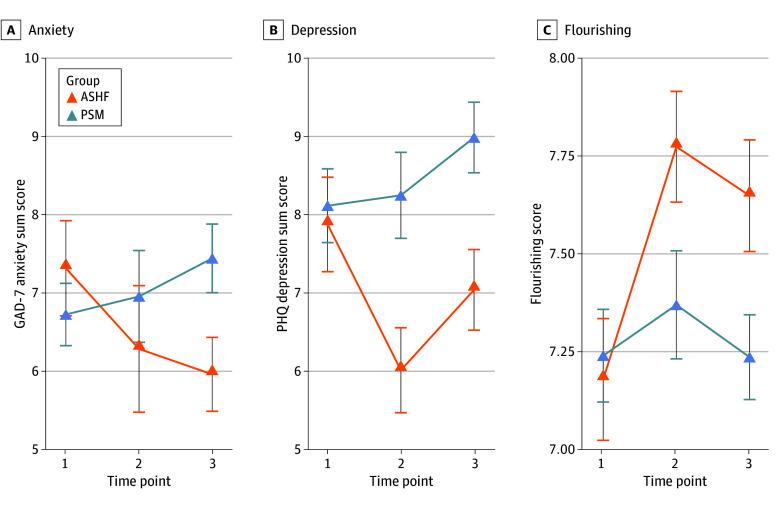
Longitudinal Trajectories of Anxiety, Depression, and Flourishing by Group The figure shows anxiety symptom scores (A), depressive symptom scores (B), and flourishing scores (C). Descriptive data are plotted. Error bars are SE of the mean. The x-axis is time: time point 1 is baseline; time point 2 is posttest (ie, the end of the semester during which baseline was assessed). Data from time point 2 are not used in the statistical models reported in this article; time point 3 is collected during the SARS-CoV-2 lockdown in Spring 2020. ASHF indicates Art and Science of Human Flourishing course participants; GAD-7, Generalized Anxiety Disorder 7-Item; PHQ, Patient Health Questionnaire; PSM, propensity score–matched control participants.

## Discussion

In this prospective cohort study of a college course about flourishing, prior participation in the course was associated with reduced anxiety and depressive symptoms, and increased flourishing during the SARS-CoV-2 lockdown. The control group mean on the Patient Health Questionnaire 8 (PHQ-8) was within the optimal screening range for major depressive disorder whereas ASHF mean PHQ-8 scores were around 12% below the cutpoint. ASHF outcomes were not significantly associated with duration since the course ended.

Although the ASHF is a promising scalable mental health intervention, this study’s results are preliminary and subject to limitations. Causal inference in PSM designs requires strong assumptions. For example, we cannot be sure that covariate adjustments controlled for unobserved differences between the groups or responders and/or nonresponders. Additionally, we sampled from 3 US colleges that are not representative of all college environments or college students. Despite these limitations, these data illustrate the potential benefits of innovative coursework during a critical developmental transition.

## Notes

Supplement 1.
eAppendix

Supplement 2.
Data Sharing Statement
