# Supplementary material for: College Course About Flourishing and Students’ Mental Health During SARS-CoV-2
Source: JAMA Netw Open. 2024 Nov 13;7(11):e2444845. doi: 10.1001/jamanetworkopen.2024.44845 (PMC11561689; doi:10.1001/jamanetworkopen.2024.44845)
Supplement: Supplement 1. — eAppendix [file jamanetwopen-e2444845-s001.pdf]

Supplemental Online Content

Hirshberg MJ, Colaianne BA, Greenberg MT, et al. College course about flourishing and students' mental health during SARS-CoV-2. *JAMA Netw Open.* 2024;7(11):e2444845. doi:10.1001/jamanetworkopen.2024.44845

eAppendix. Methods
eReferences

This supplemental material has been provided by the authors to give readers additional information about their work.

© 2024 Hirshberg MJ et al. *JAMA Network Open.*

eAppendix

Methods

Measures

Overall attention function was assessed with the 10-item Attentional Function Index.[1] Attention regulation was assessed with three items from the Attention Regulation subscale of the Multidimensional Assessment of Interoceptive Awareness.[2] Common humanity was assessed with the 4-item subscale of the same name from the Piedmont Spiritual Transcendence Scale.[3] Compassionate roommate goals were measured with the 9-item Compassionate Goals scale.[4] Empathic concern (3-items) and perspective-taking (4-items) were assessed with the respective subscales from Interpersonal Reactivity Index.[5] Healthy emotionality was assessed using the 24-item Emotional Styles Questionnaire.[6] Mindfulness was assessed with a 24-item version of the Five Facet Mindfulness Questionnaire.[7] Search for meaning was measured using the same named subscale of the Meaning in Life Questionnaire.[8] Self-compassion was assessed with the 10-item Self-Compassion Short Form.[9] Social awareness was assessed with the Social Awareness Index.[10] Stress was assessed with a single item from the American College Health Questionnaire.[11] Sleep was assessed with five-items from the Pittsburgh Sleep Quality Index.[12]

Statistical Analyses

The MICE package[13] in R was used to conduct multiple imputation by chained equations. All baseline variables included in Table 1 as well as follow-up outcomes (i.e., anxiety, depression

© 2024 Hirshberg MJ et al. *JAMA Network Open*.

and flourishing) were included in the procedure, along with group (PSM control, ASHF), race, gender, university and cohort. Maximum iteration was set to 5 with the seed set to 1988 for replicability. We examined the 50 imputed datasets for convergence and viability by plotting imputed distributions against the observed distributions, traceplots and checking value ranges. We then estimated a mixed effects model that regressed the outcome at follow-up onto group (PSM control, ASHF), gender (Woman, Man), race (African American, Asian/Pacific Islander, Hispanic, Middle Eastern combined; White), the following baseline variables with standardized group differences ≥ |0.10|: attention function, attention regulation, mindfulness, healthy emotionality, living situation during the SARS-CoV-2 lockdown, perspective-taking, search for meaning, self-compassion, and stress; as well as baseline score on the outcome, with a random effect nesting participants within group, cohort (2018 or 2019), and university (a, b, or c; 12 level-2 clusters) to account for the hierarchal structure of our data. Results from the 50 models for each outcome were pooled according to Rubin's rules.

© 2024 Hirshberg MJ et al. *JAMA Network Open*.

eReferences

1. Cimprich B, Visovatti M, Ronis DL. The Attentional Function Index--a self-report cognitive measure. *Psychooncology*. 2011;20(2):194-202. doi:10.1002/pon.1729

2. Mehling WE, Price C, Daubenmier JJ, Acree M, Bartmess E, Stewart A. The Multidimensional Assessment of Interoceptive Awareness (MAIA). *PLOS ONE*. 2012;7(11):e48230. doi:10.1371/journal.pone.0048230

3. Piedmont RL. Does Spirituality Represent the Sixth Factor of Personality? Spiritual Transcendence and the Five-Factor Model. *J Pers*. 1999;67(6):985-1013. doi:10.1111/1467-6494.00080

4. Crocker J, Canevello A. Creating and undermining social support in communal relationships: the role of compassionate and self-image goals. *J Pers Soc Psychol*. 2008;95(3):555.

5. Davis MH. *Interpersonal Reactivity Index*. Edwin Mellen Press; 1980.

6. Kesebir P, Gasiorowska A, Goldman R, Hirshberg MJ, Davidson RJ. Emotional Style Questionnaire: A multidimensional measure of healthy emotionality. *Psychol Assess*. 2019;31(10):1234-1246. doi:10.1037/pas0000745

7. Baer RA, Smith GT, Lykins E, et al. Construct validity of the five facet mindfulness questionnaire in meditating and nonmeditating samples. *Assessment*. 2008;15(3):329-342.

8. Steger MF, Frazier P, Oishi S, Kaler M. The meaning in life questionnaire: Assessing the presence of and search for meaning in life. *J Couns Psychol*. 2006;53(1):80.

9. Raes F, Pommier E, Neff KD, Van Gucht D. Construction and factorial validation of a short form of the self-compassion scale. *Clin Psychol Psychother*. 2011;18(3):250-255.

10. Lerner RM, Roeser RW, Phelps E. *Positive Youth Development & Spirituality: From Theory to Research*. Templeton Foundation Press West Conshohocken, PA; 2008.

11. American College Health Association. American College Health Association-National College Health Assessment II. Published online 2013.

12. Buysse DJ, Reynolds CF, Monk TH, Berman SR, Kupfer DJ. The Pittsburgh sleep quality index: A new instrument for psychiatric practice and research. *Psychiatry Res*. 1989;28(2):193-213. doi:10.1016/0165-1781(89)90047-4

© 2024 Hirshberg MJ et al. *JAMA Network Open*.

13. Buuren S, Groothuis-Oudshoorn K. mice: Multivariate imputation by chained equations in R. *J Stat Softw*. 2011;45(3). Accessed March 2, 2017. http://doc.utwente.nl/78938/

14. Rubin DB. *Multiple Imputation for Nonresponse in Surveys*. Vol 81. John Wiley & Sons; 2004.

© 2024 Hirshberg MJ et al. *JAMA Network Open*.
