# Supplementary material for: College Course About Flourishing and Students’ Mental Health During SARS-CoV-2
Source: JAMA Netw Open. 2024 Nov 13;7(11):e2444845. doi: 10.1001/jamanetworkopen.2024.44845 (PMC11561689; doi:10.1001/jamanetworkopen.2024.44845)
Supplement: Supplement 2. — Data Sharing Statement [file jamanetwopen-e2444845-s002.pdf]

Data Sharing Statement

Hirshberg. College Course About Flourishing and Students' Mental Health During SARS-CoV-2. *JAMA Netw Open*. Published November 13, 2024.
doi:10.1001/jamanetworkopen.2024.44845

Data
Data available: No

Additional Information
Explanation for why data not available: Due to complications arising from multiple IRBs, data from this study cannot be made publicly available.
